# Targeting secretory leukocyte protease inhibitor (SLPI) inhibits colorectal cancer cell growth, migration and invasion via downregulation of AKT

**DOI:** 10.7717/peerj.9400

**Published:** 2020-07-14

**Authors:** Zhijiang Wei, Guiying Liu, Rufu Jia, Wei Zhang, Li Li, Yuanyuan Zhang, Zhijing Wang, Xiyong Bai

**Affiliations:** 1 The First Department of Surgical Oncology, Cangzhou Central Hospital, Cangzhou, China; 2 The Brain Science Unit, CangZhou Central Hospital, Cangzhou, China

**Keywords:** Secretory leukocyte protease inhibitor, Colorectal cancer, AKT

## Abstract

The secretory leukocyte protease inhibitor (SLPI) is a serine protease inhibitor which plays important role in bacterial infection, inflammation, wound healing and epithelial proliferation. Dysregulation of SLPI has been reported in a variety of human cancers including glioblastoma, lung, breast, ovarian and colorectal carcinomas and is associated with tumor aggressiveness and metastatic potential. However, the pathogenic role of SLPI in colorectal cancer is still unclear. Here we showed that SLPI mRNA level was significantly upregulated in colorectal cancer tissues compared to adjacent normal controls. Targeting SLPI by siRNA inhibited proliferation, migration and invasion of colorectal cancer cells lines HT29 and HT116 in vitro. Mechanistically, blockage of cancer cell growth and metastasis after SLPI knockdown was associated with down-regulation of AKT signaling. In conclusion, SLPI regulated colorectal cell growth and metastasis via AKT signaling. SLPI may be a novel biomarker and therapeutic target for colorectal cancer. Targeting AKT signaling may be effective for colorectal cancer treatment.

## Introduction

Colorectal cancer (CRC) is one of the most widespread malignancies and is the second leading cause of cancer-related mortality (Parkin, 2001; Caravati-Jouvenceaux et al., 2011). Although the survival rate for colorectal cancer patients have risen over the last few years, approximately 40% ± 10% of patients suffered from disease relapse or metastasis (Matsuda et al., 2016; Pedrazzani et al., 2016).

The secretory leukocyte protease inhibitor (SLPI) is a ∼11.7-kDa, non-glycosylated, single-chain protein, which is expressed by a wealth of cell types, including secretory cells of the salivary glands, lung epithelial cells, and multiple host inflammatory and some immune cells  (Camper et al., 2016; Habgood et al., 2016; Klimenkova et al., 2014; Majchrzak-Gorecka et al., 2016; Weldon et al., 2007). Recent studies showed that SLPI is also upregulated in several human cancers including lung, cervix, ovarian, pancreas and head/neck cancers (Treda et al., 2014; Cordes et al., 2011), and is associated with cancer progression, metastasis and invasion (Rasool et al., 2010). Besides that, it has been reported that SLPI was up-regulated in colon cancer tissues when comparing to normal mucosa (Gu et al., 2013), and correlated with differentiation grade, TNM (tumor, node, metastasis) stages, lymph node metastasis and distant metastasis.

The phosphatidylinositide-3-kinase (PI3K)/protein kinase B (AKT) pathway is critical in the development of various solid tumors (Barra et al., 2018; Lei et al., 2018; Sun et al., 2015; Papadimitrakopoulou, 2012). Of note, activation of the PI3K/AKT pathway correlated with prognosis in stage II colon cancer (Malinowsky et al., 2014). However, the regulation between SLPI and AKT pathway has not been reported.

In the present study, SLPI was found to be highly expressed at the mRNA level in colorectal cancer tissues. SLPI knockdown by siRNA effectively reduced the proliferation and metastasis of colorectal cancer cells in vitro. The reduction of cancer growth was associated with the downregulation of AKT. Indeed, targeting AKT signaling by siRNA also significantly inhibited tumor cell growth, migration and invasion, suggesting that AKT is the downstream of SPLI and may be a promising therapeutic target in colorectal cancer with high SLPI expression.

## Material and Methods

### Cell culture and primary samples

The American Type Culture Collection (ATCC) (Manassas, VA) provided the human CRC cell lines (HT29 and HT116). Cells were cultured in DMEM/F-12 containing 2.2 g/L sodium bicarbonate, 0.2 g/L BSA, 50 mL/L FBS and 10 mL/L 100 units/ml penicillin, 100 µg/ml streptomycin antibiotic and antimycotic solution under the condition of 5% CO_2_ at 37 °C. After 2 months, all cell lines were discarded, and new lines were propagated from the freezer. CRC cancer tissues and matched juxtacancerous normal colorectal tissues were obtained from 60 patients with a median age of 65 years (range 40–90 years) and no history of radiation and chemotherapy at our hospital. All these tissues were histologically verified with hematoxylin and eosin staining by a pathologist. Liquid nitrogen was applied for all tissues to snap-freeze for further use. Verbal informed consent was obtained in all cases. All clinical data were analyzed anonymously. The CangZhou Central Hospital granted Ethical approval to carry out the study within its facilities.

### siRNA-mediated gene knockdown

Cell Signaling Technology provided siAkt (No. 6211). The siRNAs for SLPI was designed by online software (http://rnaidesigner.invitrogen.com/) and shown in Table 1. SLPI siRNA and control siRNA fragments were synthesized by Shanghai Sangon (Shanghai, China). Briefly, CRC cells were transfected with these siRNAs at the concentration of 300 pmol each well according to the instructions of Lipofectamine 2000 (Invitrogen). Media was exchanged 4 h after transfection and replaced with DMED medium supplemented withFBS. Media was again exchanged with fresh media at 36 h and cells were counted at 72 h.

**Table 1:** The sequences of four siRNAs for SLPI knockdown assay.

**Gene**	**siRNA number**	**Sequences**
SLPI	S1	5′-AAGCTGGAGTCTGTCCTCCTAAGAA-3′
SLPI	S2	5′-CAGTGCAAGCGGTGACTTGAAGTCTT-3′
SLPI	S3	5′-TCAAAGCTGGAGTCTGTCCTCCTAA-3′
SLPI	S4	5′-CAAAGCTGGAG TCTGTCCTCCTAAG-3′

### MTT assay

MTT assay was performed to measure cell viability. Briefly, cell suspension was prepared at a density of 5 × 10^5^ cells/mL and seeded into a 96-well plate. After culturing at 37 °C with 5% CO_2_ for 12 h, MTT (20 µL/well) was added to each well and incubated for another 4 h. Subsequently, 150 µL dimethylsulfoxide (DMSO) was added to dissolve the crystals sufficiently for scrolling 30 min at 37 °C. The OD value was quantified at 570 nm using a Universal Microplate Spectrophotometer (Infinite F50; Tecan, Männedorf, Switzerland).

### Migration assay

The transfected cells were collected and prepared at a density of 4 × 10^5^ cells/mL. Then, 100 µL cell suspension (4 × 10^5^ cells/well) was added to upper wells. Growth media were placed into lower wells and cells were incubated at 37 °C for 6 h. Migrated cells were fixed with methanol for 10 min and stained with Mayer’s hematoxylin (DakoCytomation, Glostrup, Denmark). Photomicrographs of five random fields were taken (Olympus CK2; X100), and cells were then counted using a NIH image J program (NIH Image; Bethesda, MD).

### Invasion assay

Cell invasion was determined using the Boyden Chamber assay described by Ishizu (Huynh et al., 2010) with the following modifications. Chamber inserts were pre-coated with BD Matrigel (BD Biosciences, San Jose, CA, USA) and medium for overnight under sterile conditions. Cells (5 × 10^5^ cells/mL) were plated into the upper chambers and cultured for 24 h at the atmosphere of 37 °C and 5% CO_2_. Cells in the upper chambers were removed before the membranes were fixed and stained with Quick-Dip (Fronine, Sydney, Australia). The migrated cells were counted from 24 random fields at a 40X magnification using a NIKON Coolscope (Coherent Scientific, Adelaide, Australia).

### Quantitative real-time PCR (qRT-PCR)

RNA was extracted from colorectal cancer tissues, matched juxtacancerous normal colorectal tissues, cultured SLPI, and control siRNA cells using TRIzol regent. The RNA purity was determined by ultraviolet spectrophotometer. Total RNA (1.0 µg) was converted into cDNA using a reverse transcription kit (Fermentas China Co., Ltd, Beijing, China). The qPCR reaction was performed using a SYBR Green master kit (Applied Biosystems, USA). 1 µl cDNA was used as template in a 10 µl system. The primers of SLPI and GAPDH were shown in Table 2. The thermocycler condition is: 95 °C pre-denaturing for 30 s, followed by 40 cycles of 95 °C for 30 s, annealing temperature of 60 °C for 30 s. The melting curve was made after the amplification. Each sample was repeated in triplicate. The relative expression of SLPI was calculated by ΔΔCt method, and the expression level was calculated as 2-ΔΔCt. Each value was normalized against that of GAPDH mRNA.

**Table 2:** The sequences of primers for the SLPI and GAPDH qRT-PCR analysis.

**Gene**	**Sequences**	
SLPI	Forward	5′-CCCTTCCTGGTGCTGCTT-3′
SLPI	Reverse	5′-CCTCCTTGTTGGGTTTGG-3′
GAPDH	Forward	5′-AGGCTGTGGGCAAGGTCA-3′
GAPDH	Reverse	5′-CGTCAAAGGTGGAGGAGTGG-3′

### Western =blotting

Total protein was extracted form CRC tumor tissues, corresponding adjacent normal tissues and cultured cells. The protein concentration was determined by the BCA method. 40µg total proteins were separated by SDS-PAGE, followed by transferring to a PVDF membrane (GE Healthcare). Then the membranes were blocked with 5% skimmed milk in Tris-buffered saline (TBS) containing 0.1% Tween-20 (TBS-T), for 1 h and subsequently incubated overnight at 4 °C with the primary antibodies, including SLPI (#sc373802, 1:200, Santa Cruz biotechnology, Santa Cruz, CA), Phospho-AKT (#9271, 1:1000, Cell Signaling Technology) and AKT (#9272, 1:1000, Cell Signaling Technology), and GAPDH (1:1000, #sc32233, Santa Cruz Biotechnology, Santa Cruz, CA). After that, the membrane was incubated with horseradish peroxidase-conjugated secondary antibodies IgG (Sigma, 1: 5,000) for 2 h at room temperature. The protein bands were visualized with Amersham ECL substrates and analyzed by Multigauge computer software (Berthold, Bundoora, Australia).

### Statistical analysis

All data was shown as mean ± SD and analyzed with SPSS 13.0 (SPSS, Chicago, IL). One-way ANOVA with Bonferroni’s correction was used to compare the difference among three or more groups. The comparison between groups using LSD test. For all comparisons, differences were considered as statistically significant when *p* < 0.05. The serum markers for CRC was evaluated by the empirical receiver operating characteristic (ROC) curve.

## Results

### SLPI was upregulated in colorectal cancers compared to adjacent normal tissues

**Figure 1:**
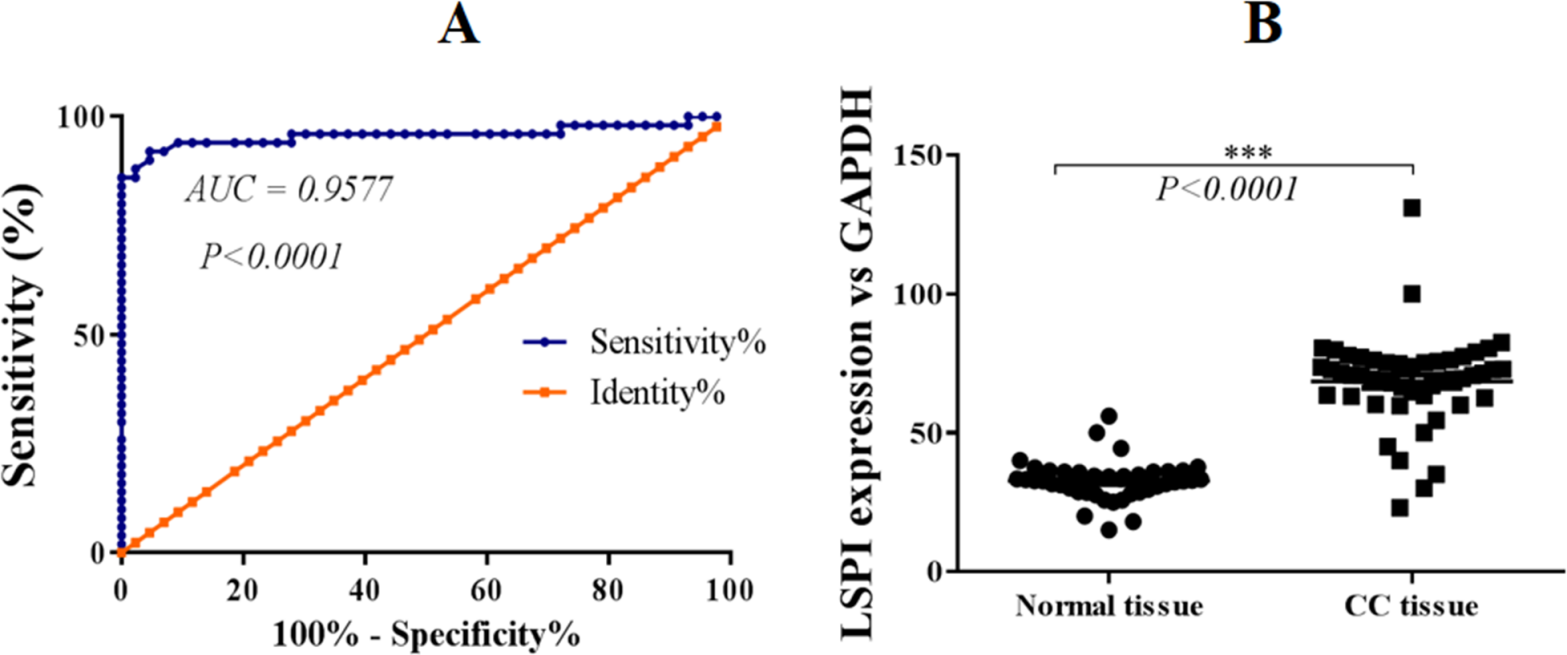
SLPI is upregulated in colorectal cancers. (A) ROC curve analysis of the expression of SLPI in colorectal cancers. (B) Detection of SLPI mRNA expression by qRT-PCR in normal and colorectal cancer tissues.

SLPI has been reported to be upregulated in a variety of cancers and plays important role in metastasis of cancer cells. However, the expression and pathogenetic role of SLPI in colorectal cancer remains elusive. Here we showed that SLPI transcript level was significantly increased in our archived primary colorectal cancer samples when comparing to normal tissues (Fig. 1A).

Further, ROC curves were utilized to evaluate the performance of SLPI in discriminating colorectal tissue from adjacent normal tissues. The area under the curve (AUC) was 95.77% for colorectal cancer detection (Fig. 1B). Sensitivity ranged from 86.5 to 100% and specificity was 100%, indicating a statistically significant correlation between SLPI and colorectal cancers (*p* < 0.0001).

### SLPI knockdown by siRNA suppressed the proliferation, migration and invasion of colorectal cancer cells

**Figure 2:**
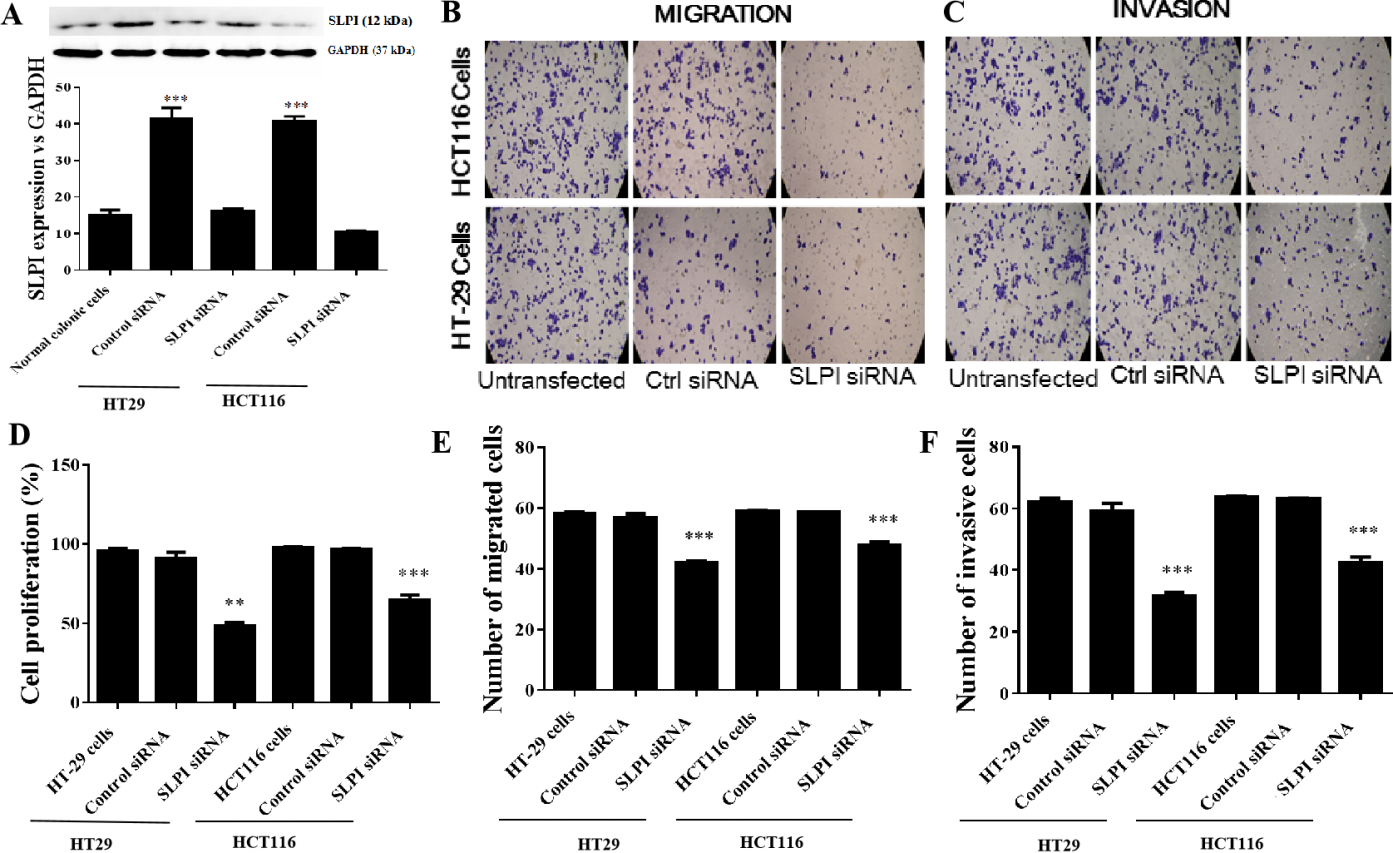
Effects of SLPI knockdown on the proliferation, migration and invasion of colorectal cancer cells. (A) Detection and quantitative analysis of SLPI protein expression after siRNA knockdown in colorectal cancer cell lines HT-29 and HCT116 by Western blotting. The untransfected (normal) and control siRNA-transfected cells were used as controls. Proliferation (D), migration (B,E) and invasion (C,F) of both cell lines were significantly inhibited by siRNA-mediated SLPI knockdown.

As SLPI was significantly upregulated in colorectal cancer tissues, we sought to determine whether SLPI was essential for the survival of colorectal cancer cells. Here, SLPI was knocked down by siRNA in colorectal cancer cell lines HT29 and HT16. Four different siRNAs were designed and one of them showed the strongest knockdown effect by qRT-PCR. Consistently, SLPI expression was significantly decreased in HT29 and HT116 cells after SLPI knockdown compared to control siRNA transfected cells (Fig. 2A). In addition, proliferation (Fig. 2D), migration (Figs. 2B and 2E), and invasion (Figs. 2C and 2F) were also significantly reduced after SLPI knockdown in HT29 and HT116 cells compared to the control siRNA group. These data revealed that SLPI may play important roles in the survival, migration and invasion of colorectal cancer cell in vitro.

### SLPI knockdown reduced the phosphorylation of AKT

**Figure 3:**
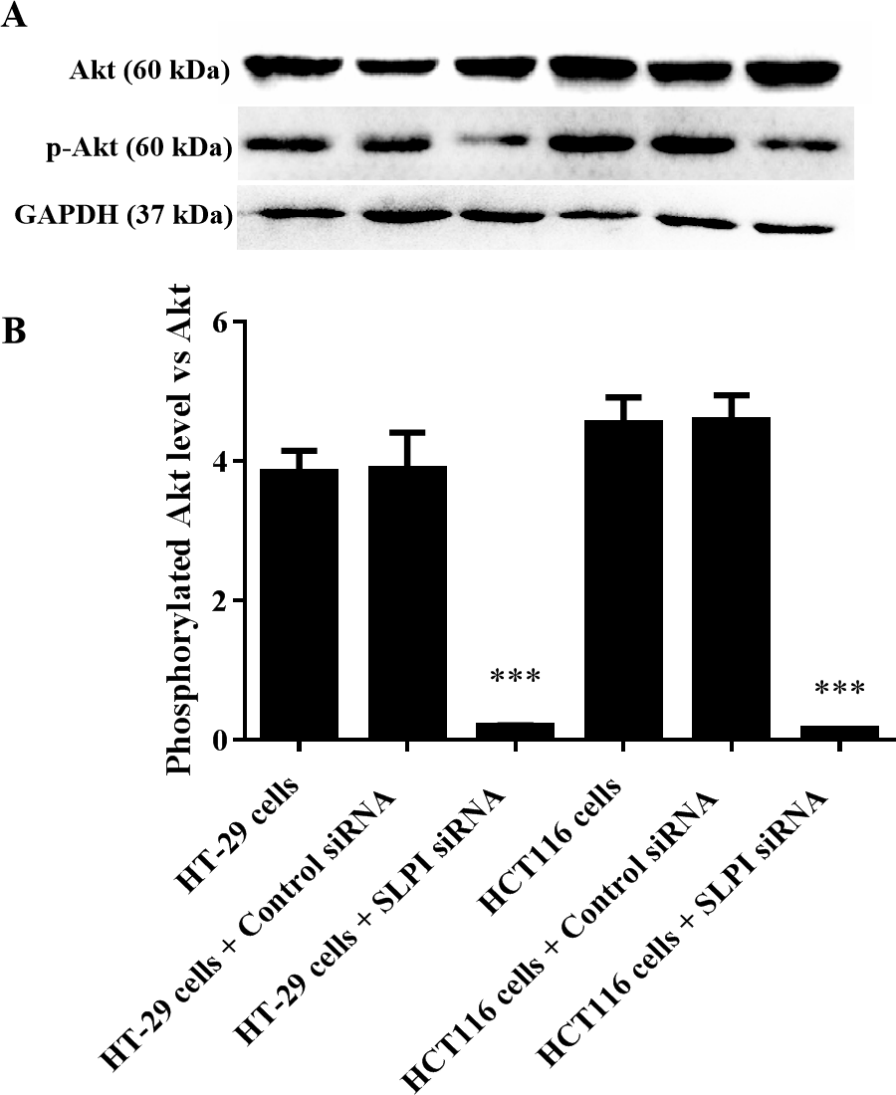
Effect of SLPI knockdown on the activation of AKT in colorectal cancer cells. (A) Total and phosphorylated AKT were detected by Western blotting after SLPI knockdown in HT-29 and HCT116 cells, respectively. (B) The relative expression of total and phosphorylated AKT was quantified by the intensity of the bands by ImageJ.

AKT kinases are involved in a variety of cellular processes including cell proliferation, survival, and metastasis. Next, we sought to investigate the potential crosstalk between SLPI expression and AKT activation in colorectal cancers. Total AKT was not affected after SLPI knockdown in HT-29 and HT-116 cells (Fig. 3A). Whereas, the phosphorylated AKT, an active form of AKT, was markedly decreased compared to the control siRNA-transfected cells (Figs. 3A and 3B). These data suggested that the activation of AKT was regulated by SLPI in colorectal cancer cell lines.

### AKT as a promising therapeutic target for colorectal cancers

**Figure 4:**
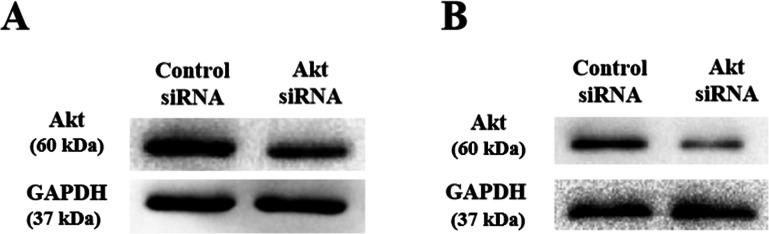
Detection of AKT protein expression after siRNA knockdown in colorectal cancer cell lines HT-29 and HCT116 by Western blotting. (A) Expression of AKT and GAPDH (loading control) in HT-29 AKT knockdown and control cells. (B) Expression of AKT and GAPDH (loading control) in HCT116 AKT knockdown and control cells.

**Figure 5:**
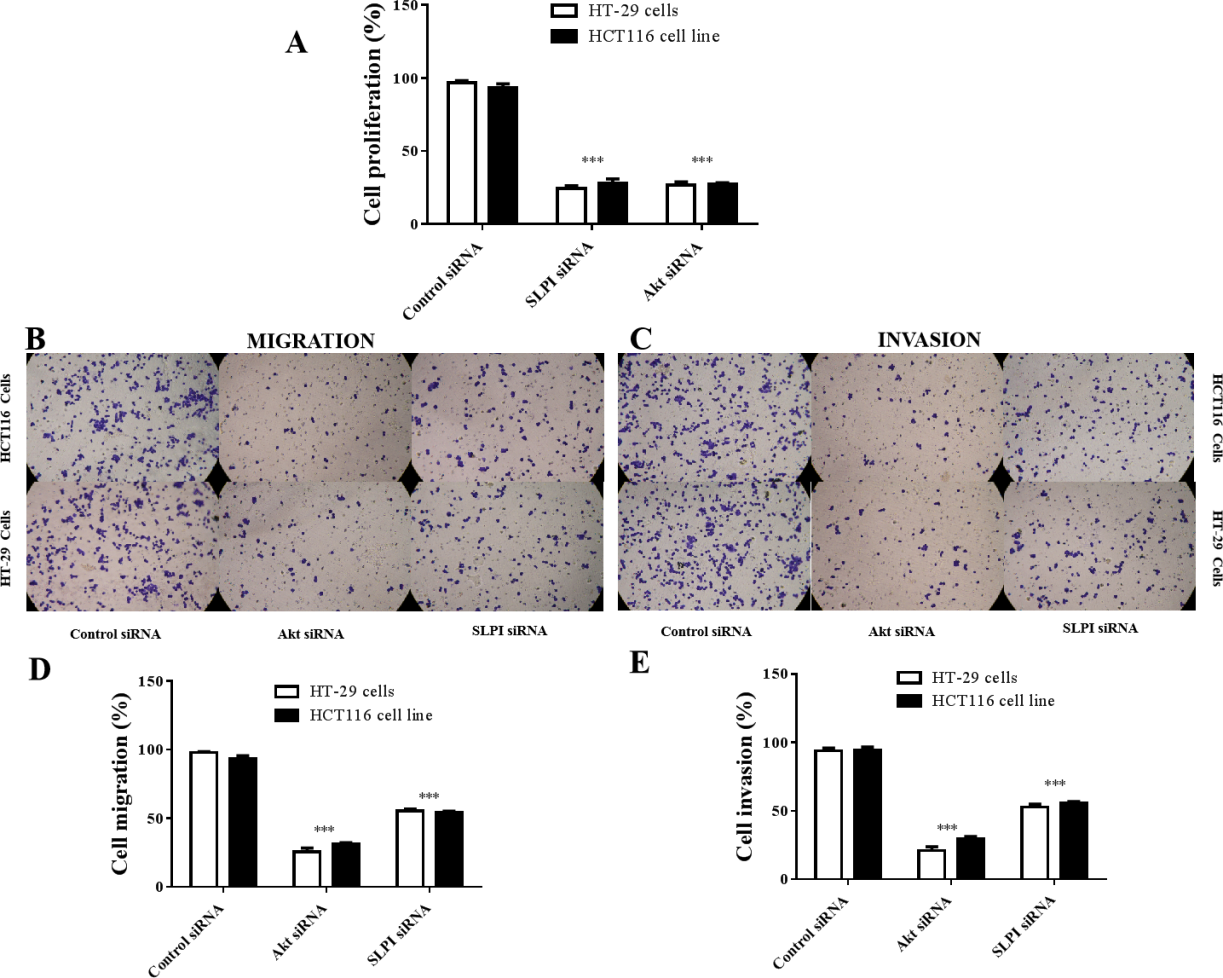
Effect of siRNA-mediated AKT knockdown on the survival, migration and invasion of colorectal cancer cell lines HT29 and HCT116 Proliferation. (A) Migration (B, D) and invasion (C, E) of both cell lines were measured and quantified after SLPI or AKT knockdown, respectively.

As shown above, SLPI knockdown suppressed the survival and invasive potential of colorectal cancer cells via downregulation of AKT. It will be interesting to test whether targeting AKT could be promising therapeutics for colorectal cancer. We showed that AKT was effectively knocked down at the protein level by siRNA as demonstrated by Western blotting (Figs. 4A and 4B). Importantly, proliferation (Fig. 5A), migration (Figs. 5B and 5D) and invasion (Figs. 5C and 5E) of HT29 and HT116 cells were significantly reduced following siRNA-mediated AKT knockdown, suggesting that targeting AKT may be effective for treatment of colorectal cancers with high SLPI expression. Collectively, these data demonstrated that the AKT pathway was regulated by SLPI and targeting AKT may be effective for the treatment of colorectal cancer.

## Discussion

In the present study, we first demonstrated that SLPI was significantly increased in colorectal cancers. SLPI was originally isolated from respiratory secretions from patients with chronic obstructive pulmonary disease (COPD) and downregulation of SLPI was strongly associated with the incidence of COPD (Nukiwa et al., 2008). It has been reported that SLPI is upregulated in non-small cell lung cancer (NSCLC), and cancers of the cervix, ovary, and pancreas, but not in those of the kidney, intestinal tract, breast, or nasopharynx (Bouchard et al., 2006). Upregulation of SLPI is associated with the progression of gastric cancer and development of pancreatic ductal adenocarcinoma (PDAC) (Cheng et al., 2008). Targeting SLPI by siRNA induced apoptosis, and inhibited the migration and invasion of PDAC cells (Zhang et al., 2015). SLPI was increased in ovarian cancer and enhances the invasiveness of ovarian cancer cells via modulating MMP-9 release (Hoskins et al., 2011; Tsukishiro et al., 2005). In the previous study, SLPI was found overexpressed in colorectal cancer tissues of 296 CRC patients (Liu et al., 2014). Upregulation of SLPI is associated with tumor grade and TNM stage (tumor, node, metastasis), but not with patients’ sex or age (Cordes et al., 2011; Liu et al., 2014). In line with these findings, we showed that SLPI was consistently increased at the transcription level in public databases and our archived primary colorectal cancer samples. It is important to further investigate whether SLPI is also overexpressed the protein level by either western blotting or immunohistochemistry in our cohort, which may provide solid underpinnings for downstream functional studies.

Second, we showed that AKT was involved in SLPI-mediated pathogenesis in colorectal cancer. The exact mechanism by which SLPI promotes malignancy is still elusive. It has been proposed that SLPI inhibited elastase to promoted angiogenesis via suppression of endostatin, a potent antiangiogenic factor (Oreilly et al., 1997; Wen et al., 1999; Wright et al., 1999; Eisenberg et al., 1990; Sugino et al., 2007). SLPI also plays an important role in the regulation of cell cycle progression by promoting the expression of cyclin D1 and IGFBP-3 in tumor cells (Cheng et al., 2008; Wen et al., 2011). However, the underlying mechanism of SLPI-mediated tumor cell invasiveness remains largely unknown. It has been shown that the expression and activation of AKT are critical for cancer cell growth and proliferation (Deng et al., 2010). In this study, we showed that SLPI knockdown reduced the phosphorylation of AKT in CRC cancer cells, unveiling the potential mechanism of SLPI-mediated pathogenesis in colorectal cancers.

Last but not least, we propose that AKT may be a novel therapeutic target for colorectal cancer with high SLPI expression. Hyperactivation of AKT has been detected in human tumors with acquired chemoresistance  (Yu et al., 2008; Fan et al., 2015), including colorectal cancers resistant to cisplatin (Bellacosa et al., 2005). These observations highlight AKT as an emerging target for chemoresistance in colorectal cancer. In the present study, our data showed the AKT knockdown was more effective to suppress the migration and invasion of CRC cancer cells than those of SLPI knockdown, suggesting that AKT may be a promising and direct therapeutic target in colorectal cancers. More importantly, our present investigation unequivocally demonstrated a previously undescribed association between SLPI and AKT in the pathogenesis of colorectal cancer cells. The association between SLPI expression and disease progression should be further studied. Moreover, application of small-molecule drugs targeting AKT signal pathway should be also explored for treatment of colorectal cancers.

## Conclusions

Together, we found that SLPI transcript level was highly expressed in colorectal cancers compared to their normal counterparts. SLPI knockdown effectively reduced colorectal cancer cell growth, migration and invasion in vitro. Phosphorylation of AKT was significantly reduced after SLPI knockdown in colorectal cancer cells. Further, targeting AKT also considerably inhibited tumor cell growth, migration and invasion similar to the effects of SLPI knockdown, which indicates that AKT is the downstream of SPLI and might be a novel therapeutic target for colorectal cancer treatment with high SPLI expression. Our data will not only provide novel insights into the role of SLPI during the pathogenesis of colorectal cancer but also shed light into the potential therapeutics for colorectal cancer treatment.

##  Supplemental Information

10.7717/peerj.9400/supp-1 Supplemental Information 1 Gel/blots Figure 2 GAPDH

10.7717/peerj.9400/supp-2 Supplemental Information 2 Software used to create Gel/blot Figure 2 GAPDH

10.7717/peerj.9400/supp-3 Supplemental Information 3 Gel/blot Figure 2 SLPI

10.7717/peerj.9400/supp-4 Supplemental Information 4 Software used to create Gel/blot Figure 2 SLPI

10.7717/peerj.9400/supp-5 Supplemental Information 5 Gel/blot Figure 3 p-Akt

10.7717/peerj.9400/supp-6 Supplemental Information 6 Software used to create Gel/blot Figure 3 p-Akt

10.7717/peerj.9400/supp-7 Supplemental Information 7 Gel/blot Figure 3 GAPDH

10.7717/peerj.9400/supp-8 Supplemental Information 8 Gel/blot Figure 3 Akt

10.7717/peerj.9400/supp-9 Supplemental Information 9 Software used to create Gel/blot Figure 3 Akt

10.7717/peerj.9400/supp-10 Supplemental Information 10 Software used to create Gel/blot Figure 3 GAPDH

## Additional Information and Declarations

### Data Availability

The following information was supplied regarding data availability: The data is available at Figshare: Wei, ZJ (2020): data. figshare. Dataset. https://doi.org/10.6084/m9.figshare.11690607.v1.
