# Supplementary figures and images for: Targeting secretory leukocyte protease inhibitor (SLPI) inhibits colorectal cancer cell growth, migration and invasion via downregulation of AKT
Source: PeerJ. 2020 Jul 14;8:e9400. doi: 10.7717/peerj.9400 (PMC7367054; doi:10.7717/peerj.9400)

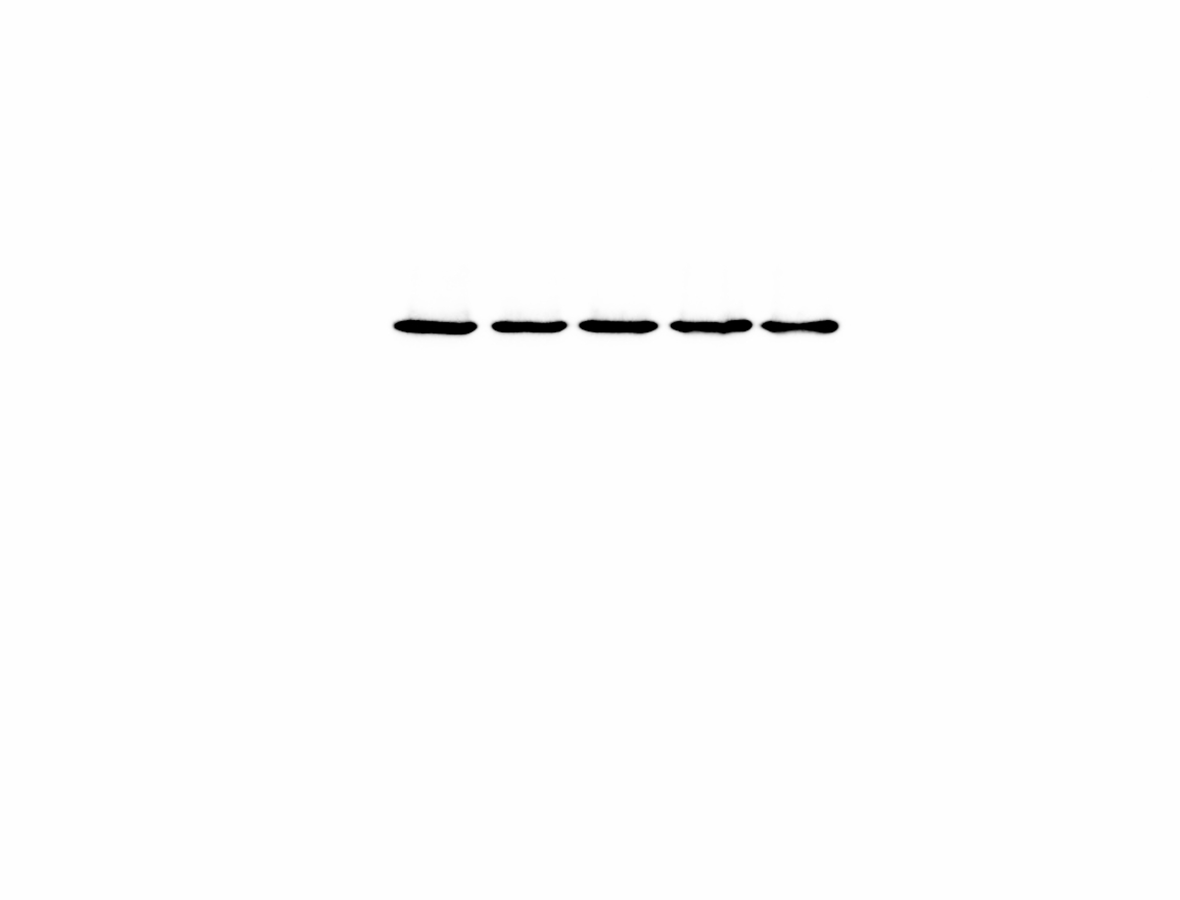

Supplement: Supplemental Information 1 [file peerj-08-9400-s001.png]

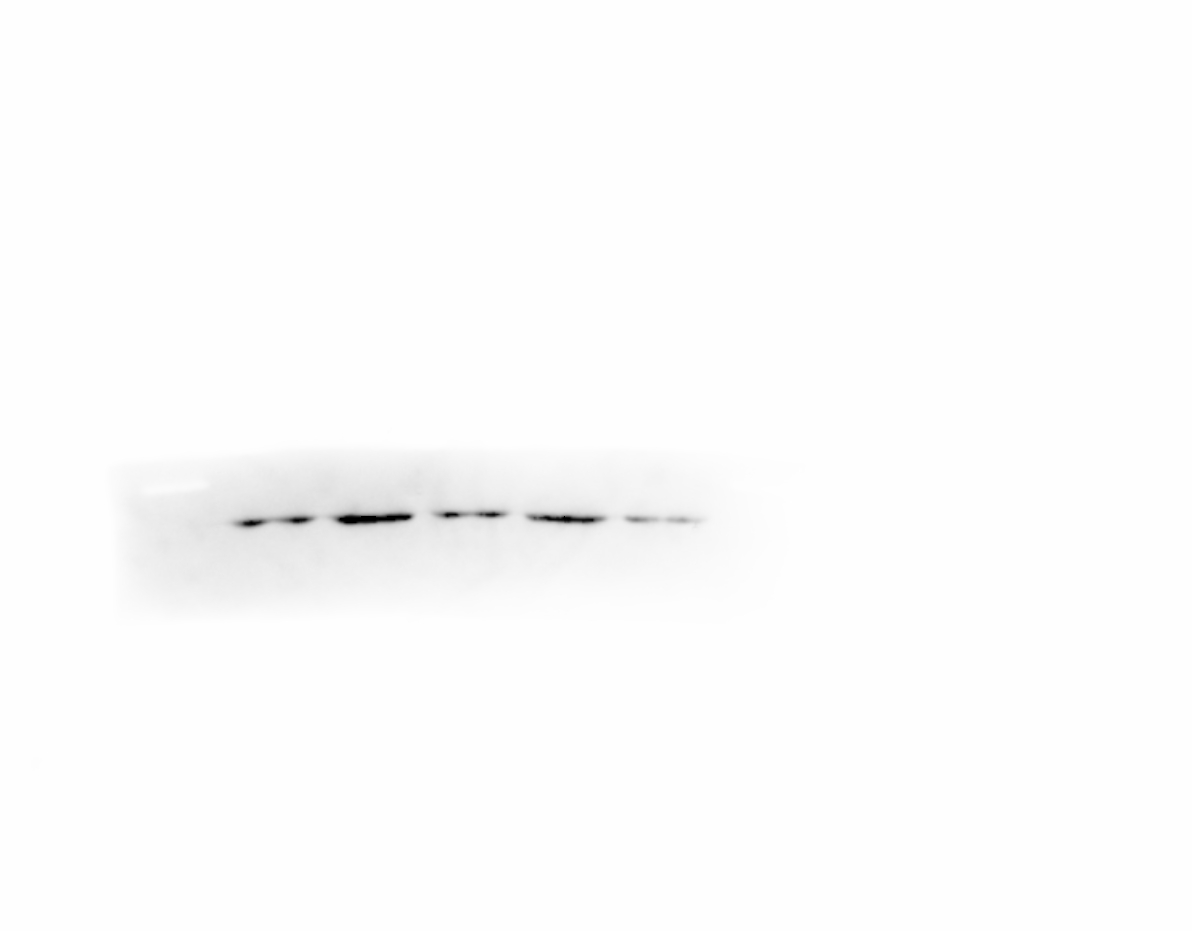

Supplement: Supplemental Information 3 [file peerj-08-9400-s003.png]

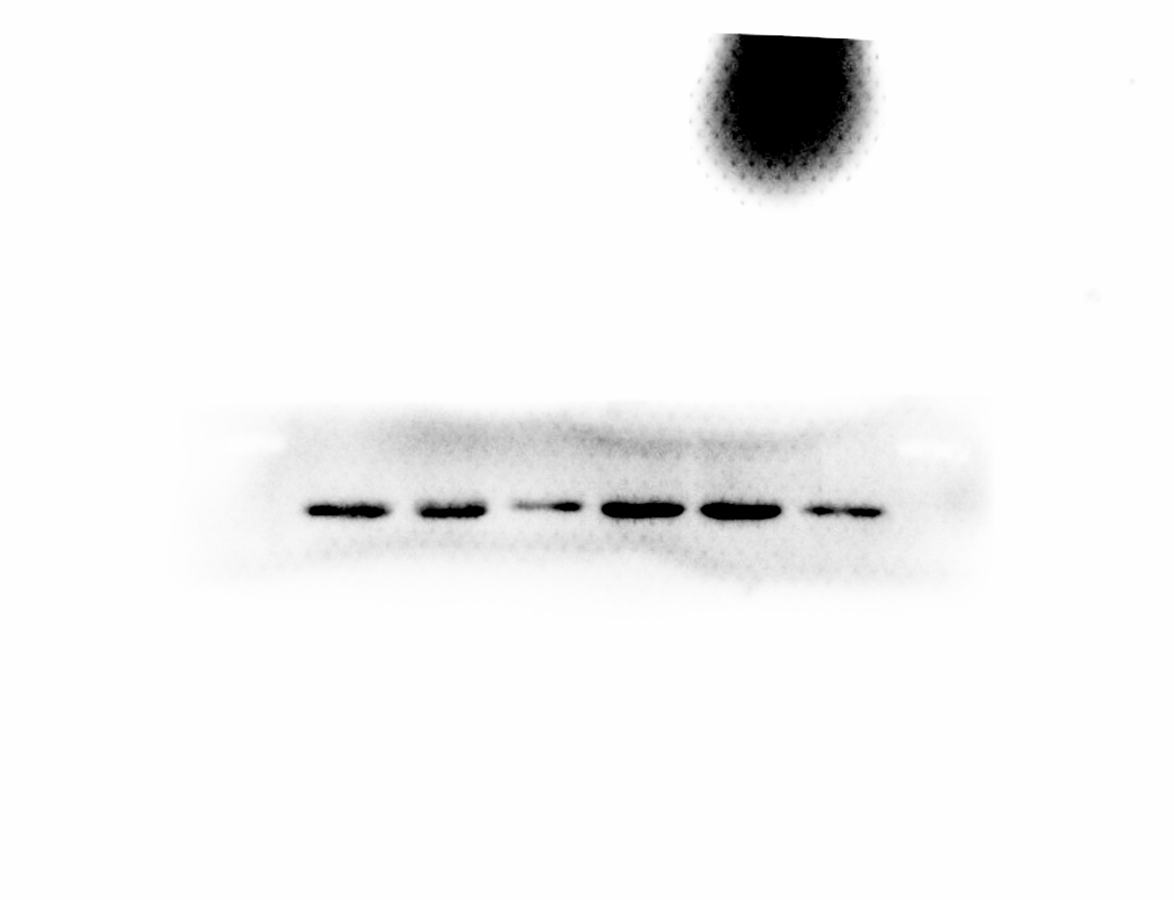

Supplement: Supplemental Information 5 [file peerj-08-9400-s005.png]

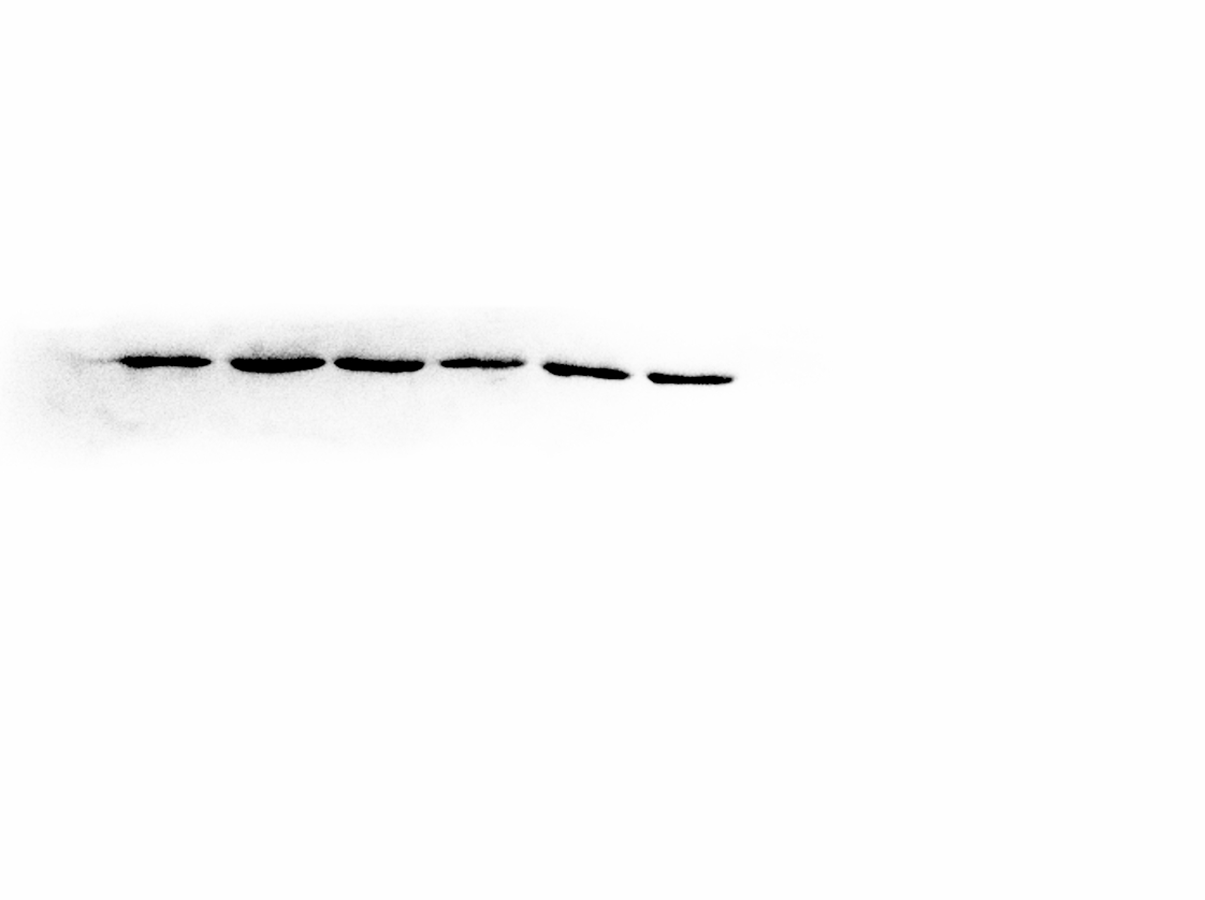

Supplement: Supplemental Information 7 [file peerj-08-9400-s007.png]

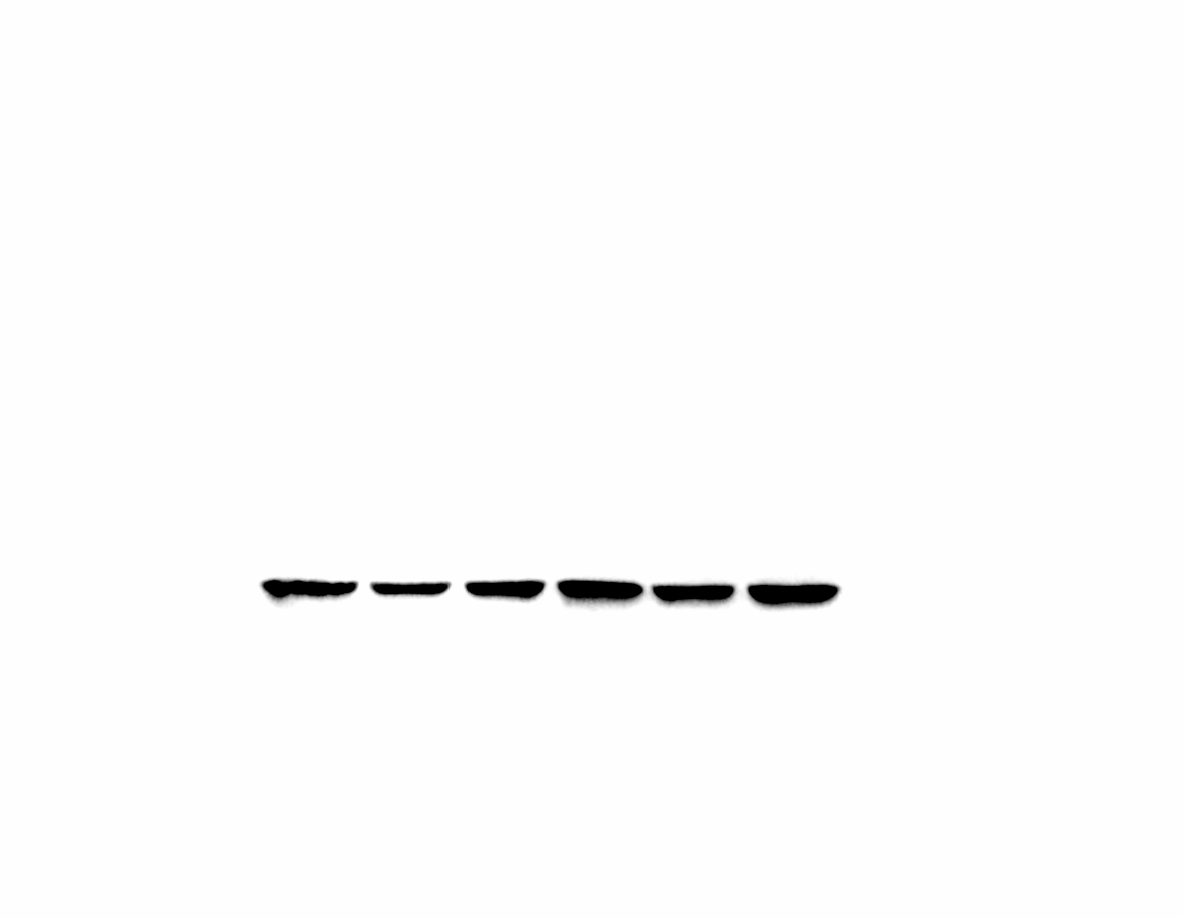

Supplement: Supplemental Information 9 [file peerj-08-9400-s009.png]
